# Minimally Invasive Cochlear Implantation: First-in-Man of Patient-Specific Positioning Jigs

**DOI:** 10.3389/fneur.2022.829478

**Published:** 2022-04-25

**Authors:** Rolf Salcher, Samuel John, Jan Stieghorst, Marcel Kluge, Felix Repp, Max Fröhlich, Thomas Lenarz

**Affiliations:** ^1^Department of Otolaryngology, Hannover Medical School, Hannover, Germany; ^2^OtoJig GmbH, Hannover, Germany; ^3^MED-EL Research Center, Hannover, Germany

**Keywords:** minimally invasive, stereotactic, frame, jig, robotic, clinical trial, image-guided surgery, cochlear implant

## Abstract

A minimally-invasive surgical (MIS) approach to cochlear implantation, if safe, practical, simple in surgical handling, and also affordable has the potential to replace the conventional surgical approaches. Our MIS approach uses patient-specific drilling templates (positioning jigs). While the most popular MIS approaches use robots, the robotic aspect is literally put aside, because our high-precision parallel kinematics is only used to individualize a positioning jig. This jig can then be mounted onto a bone-anchored mini-stereotactic frame at the patient's skull and used to create a drill-hole through the temporal bone to the patient's cochlea. We present the first clinical experience where we use sham drill bits of different diameters instead of drilling into the bone in order to demonstrate the feasibility and accuracy.

## 1. Introduction

Cochlear implant (CI) surgery is widely regarded as a success story (1). However, while CI technology has continuously improved (2), the surgical technique used in implantation has not changed significantly in its basic approach since 1961 (3). With the goal of inserting an electrode array with a diameter of maximally 1.3 mm, a transmastoid procedure with posterior tympanotomy is usually performed (4). Because this conventional procedure requires an open surgery, which is recommended to be performed only in highly specialized clinics by CI surgeons with several years of experience and training (5), it is no surprise that clinicians and researchers have investigated automation and minimally-invasive surgical (MIS) approaches. These could have the potential to overcome the disadvantages inherent in the manual procedure (6–8).

Considering the widely-used transmastoid surgical access and following the path of the electrode array insertion, it becomes clear that there is a straight line of sight from the surface of the skull through the facial recess toward the cochlea. This straight line justifies aiming for a straight minimally-invasive access path (9). In MIS, the access path has to be defined based on radiological imaging data rather than by visually exposing anatomical landmarks during open surgery. Conceptually, the step of bringing a virtually planned access path to the patient involves an image-to-patient registration and there are high accuracy requirements (10). As all relevant landmarks are embedded within the temporal bone, the skull surface is well suited to establish such necessary registration. This is usually realized either by navigational markers attached rigidly to the skull or by directly bone-anchoring a guidance apparatus to the patient's skull. The first option requires an active control loop to navigate a surgical drilling robot. The latter option is to use a passive bone-anchored stereotactic system that guides the surgical drill.

We have previously presented the accuracy and suitability of our passive, bone-anchored mini-stereotactic frame with patient-specific positioning jigs *in vitro* and *ex vivo* tests (11, 12). Herein, we present the first results of the clinical feasibility. The aims are to (1) confirm sufficient accuracy under the conditions in the operating theater and (2) investigate the suitability of our workflow. The aim of this trial, as agreed with the institutional ethics committee, is neither the minimally-invasive drilling of the access path nor the electrode insertion. We are using sham drill bits to check the accuracy similar to an approach suggested by Labadie et al. (13). Here, we report about our experience and the results from the first six out of 10 patients enrolled in this trial.

## 2. Materials and Methods

### 2.1. Description of the Clinical Trial

This clinical trial was designed as a prospective, open, interventional, monocentric, research trial, conducted at the primary sponsor Medical School Hannover (MHH) in Hannover, Germany. The study was approved by the ethics committee of MHH (vote no. 9030_BO_S_2020 from 2020-06-15), registered as DRKS00025035 at DRKS with the title “Intraoperative feasibility of patient individual positioning and guiding jigs for cochlear implantation.” A self-developed prototype system was used as described in section “System description.”

### 2.2. Inclusion and Exclusion Criteria

To be included, potential participants had to be adult CI candidates (aged 18–75 years). Exclusion criteria were revision CI surgery or having had a previous illness or condition that required a mastoidectomy or acute infection in the middle ear or the mastoid.

### 2.3. System Description

**Figure 1:**
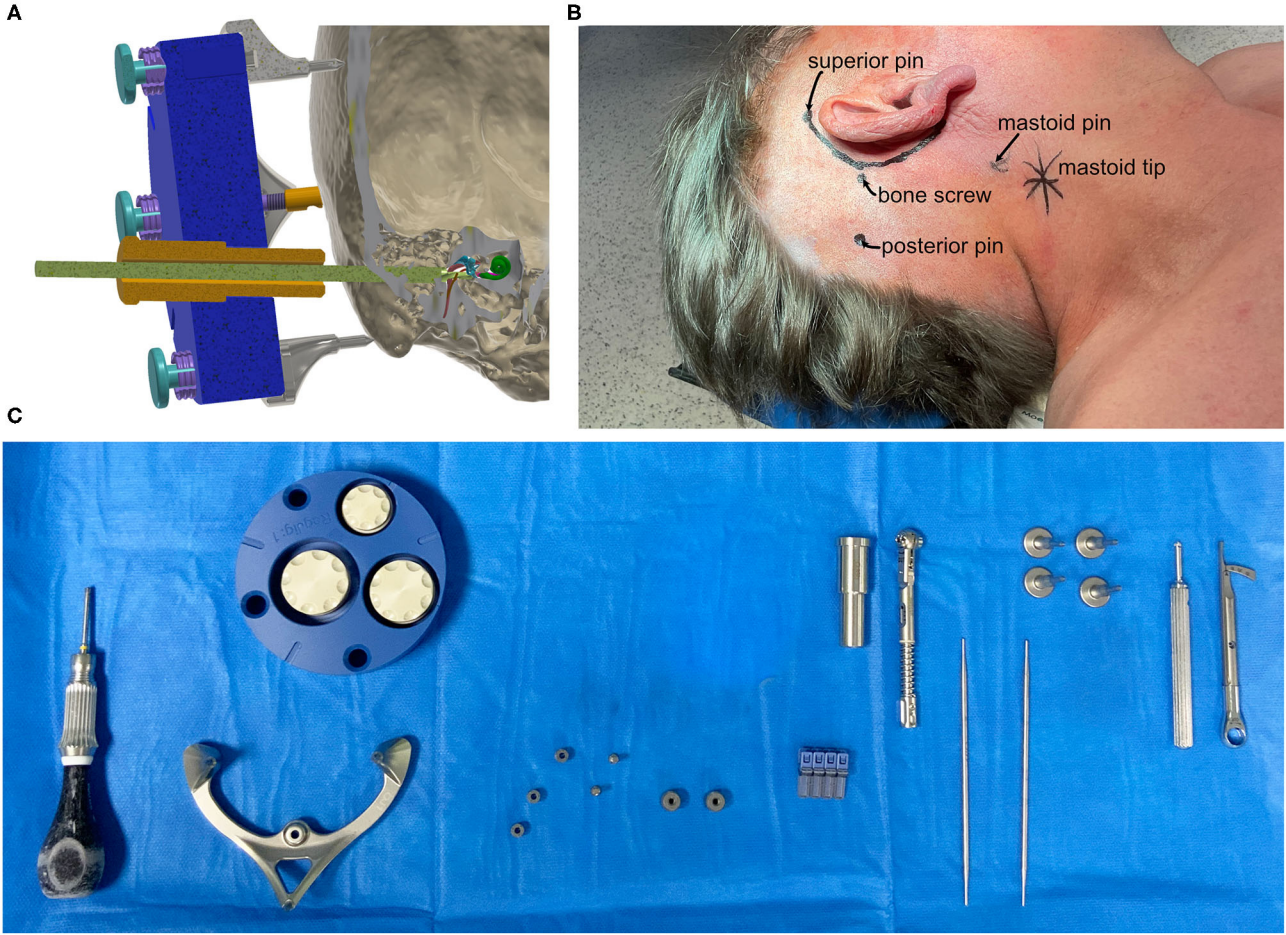
**(A)** Schematic cut-plane-rendering of the proposed mini-stereotactic frame carrying a patient-specific positioning jig (blue) with a tool guide (orange) and a drill bit (green) advanced through the temporal bone toward the entry of the cochlea. The frame and the positioning jig are connected with three jig fasteners. **(B)** The positions of the pins of the mini-stereotactic frame and the position of the bone screw indicated on a patient (case 06). **(C)** From left to right, the sterilized set in the operating theater; screw driver, mini-stereotactic frame, X-ray marker, anchor, mounting screws, bone screws, tool guide, torque ratchet, and sham drill bits, jig fastener pins, screw driver, and torque limiter.

The following components were developed and used in the trial:

- A reusable mini-stereotactic frame (frame) made of titanium (Figure 1). This frame has a C-shaped symmetrical design to fit posterior to left and right ears in adults. The frame consists of three pins which shall be in direct contact with the skull surface.
- A self-tapping bone screw (diameter 2.0 mm, length 6.0 mm, Synthes GmbH, Selzach, Switzerland), placed inside the triangular shaped area, spanned by the three pins, fixes the frame firmly on the skull in order to restrict any movements.
- An anchor and a mounting screw, both made of titanium.
- An X-ray marker made of a biocompatible polyphenylsulfone and containing multiple X-ray dense titanium balls, fixated at defined positions, can be mounted onto the frame. This X-ray marker can be automatically localized by our planning software in beam and/or computed tomography volume images (Figure 1C).
- The planning software is implemented in the Python programming language (14) as a plugin to the open source 3DSlicer imaging platform (15), mainly using the libraries NumPy (16), SciPy (17), and vtk (18). Additionally, the planning software allows automatic registration of the X-ray marker images and anatomical segmentation of the target and risk structures to be performed semi-automatically. It also allows the planning of minimally invasive access paths.
- The planned access path is then transferred to a fully automated on-site manufacturing system, which intraoperatively produces the patient-specific positioning jig. This jig receives the individual through-hole at the position and angle which corresponds exactly (12) to the already planned access path when mounted onto the frame.
- The positioning jig and the X-ray marker are mounted with three jig fasteners, which allow for simple, fast, and stable fixation. However, in case of emergency, the jig fasteners can be released quickly without any tools for full access to the sites.

Our design goal was to have the benefits from using a robot, like automation and high accuracy, without the risks of having a robot acting directly *in situ* at the patient.

### 2.4. Surgical Workflow

**Figure 2:**
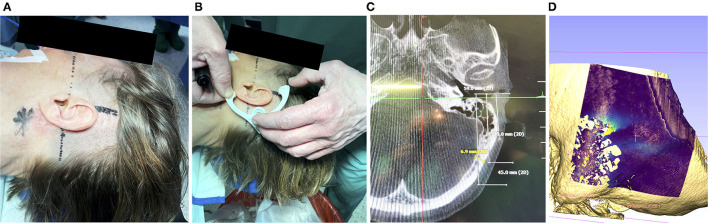
**(A)** Prior to disinfection of the surgical field, marking the mastoid tip (star shaped), drawing the orbitomeatal line (dashed) and line perpendicular (solid) can help to position the frame. **(B)** A non-sterile 3d-printed dummy frame can be useful to mark the incision points and the location where the bone screw shall be placed. **(C)** Optionally, if a navigation system has been set up, the bone thickness at the designated screw position can be documented (in this case 6.9 mm). **(D)** If a preoperative scan is available, the bone thickness can be visualized. Visible are the artifacts due to the air-filled mastoid cells and the lower bone thickness at the sigmoid sinus.

To fixate the frame onto the patient's skull, first the incision area was shaved and the position for the attachment of the frame was identified, see Figure 2A. The orbitomeatal line, passing through the outer canthus of the eye and the center of the EAC (19) can be used to position the frame, as depicted in Figure 2B. Otherwise, preoperative bone thickness measurements and planning of the optimal position with our software can further support in finding an optimal position (Figures 2C,D).

**Figure 3:**
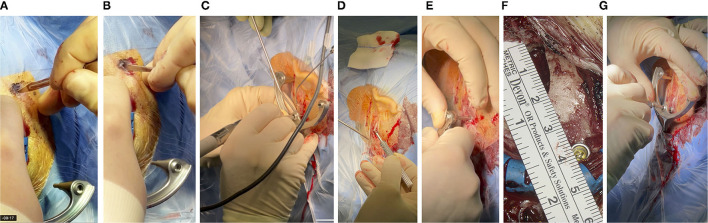
**(A)** With a thin scalpel, the skin is punctured through the foil at the three marked spots for the pins of the frame, resulting in incision lengths of about 4-6 mm. **(B)** The periost is scraped so that the pin of the frame can be pressed directly onto the skull surface. **(C)** Pre-drilling and **(D)** fixation of the anchor, with a bone screw and **(E)** toque ratchet, **(F)** about 4 cm posterior to the EAC. **(G)** The same ratchet was used to attach the frame to the skull with the mounting screw, which in turn screws onto the anchor.

Once the intended position was identified and marked, the surgical site was disinfected and covered with sterile plastic foil, following the standard OR protocol. Next, the sterile titanium frame was fixated. In a first step, a 5-7 cm incision that allows the conventional approach, was performed. If the position of the frame fixation screw did not fall within this area, an additional incision of about 1 cm was performed at the location needed. The size of the incision was measured afterward with a sterile ruler. To ensure that the pins of the frame sat directly on the skull, the skin was punctured in additional positions (Figure 3A) and the periost was removed (Figure 3B). The self-tapping bone screw requires pre-drilling. This was performed with a small 1.5 mm diamond bur (Figure 3C) and the frame was used as a template to guide the surgeon in the pre-drilling. As described above, the bone screw fixes a small anchor onto the skull. The frame is tightly fixed onto this anchor *via* the mounting screw (Figures 3D,F). For optimal stabilization, both the bone screw and mounting screw are tightened to a defined range of 30–35 Ncm (Figures 3E,G) with a torque ratchet (Josef Ganter Feinmechanik GmbH, Germany). The surgeon manually checked that the frame was firmly fixated onto the skull by carefully attempting to manually pull and rotate the assembly.

**Figure 4:**
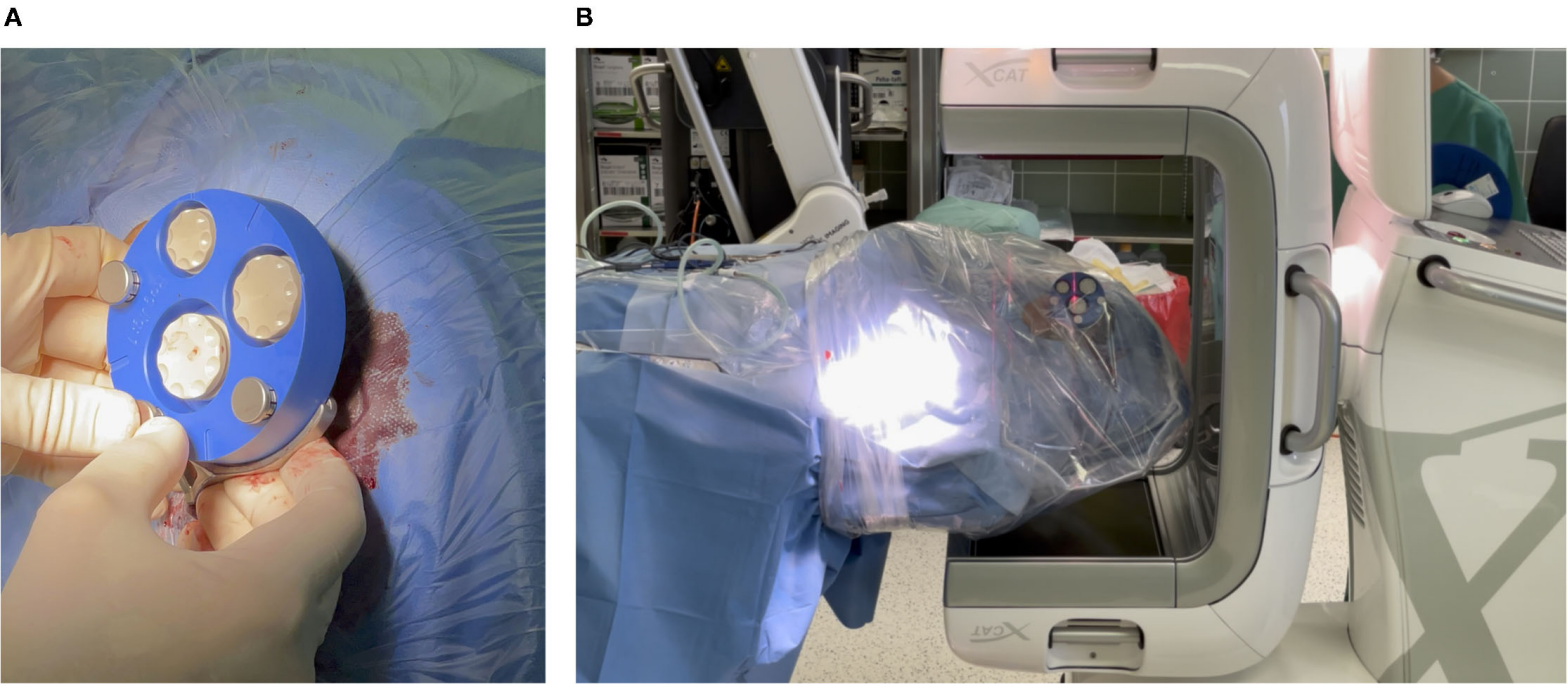
**(A)** Mounting X-ray marker (blue). The X-ray marker has dense markers embedded, which help to localize the markers in the imaging and thereby the position of the frame can be determined. **(B)** Acquiring a cone beam computed tomography (CBCT) scan. To avoid contamination, the patient's head is covered with sterile foil.

For acquiring the imaging data and planning the patient-individual access path, the X-ray marker was mounted onto the frame and fixated with three jig fasteners (Figure 4A). Then, an intraoperative cone beam computed tomography (CBCT) scan was acquired on an xCAT IQ (Xoran Technologies LLC, USA) with 0.3 mm isometric voxel size (Figure 4B). The DICOM data were exported to a USB stick.

**Figure 5:**
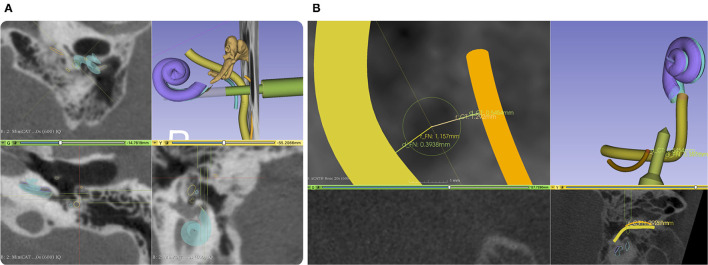
**(A)** Screenshot from our planning software. An access path (green) is planned with a diameter of 1.5 mm and 3 mm through the facial recess. The facial nerve (FN) is shown in yellow and the chorda tympani (CT) in orange. **(B)** A close-up of the projection view in the direction of the planned drill path. Here, we do not measure the width of the facial recess but the effective width referred to the central axis of the planned access path. To better compare drill diameter to the facial recess width, we report two distances, one for each nerve.

After importing the data into our planning software, an automated localization of the X-ray marker and semi-automatic segmentation of the target and risk structures were performed. The surgeon corrected and confirmed any segmentation suggestions presented by the planning software. The access path was semi-automatically planned by selecting its target at the round window. It was then visualized in 3D. The in-plane and off-plane insertion angles into the cochlea were visualized for the surgeon to further manipulate the access path and to plan a preferred trajectory for electrode array insertion, refer to Figure 5A. Furthermore, the software provided an inline view of the path to show the minimal distances to risk structures and to assess the margins. Instead of the not uniquely defined width of the facial recess, we measured the effective shortest distances from the centerline of the planned drill path to the facial nerve (FN) and the chorda tympani (CT) because those numbers can be directly compared to the radius of the drill bits (Figure 5B). A margin of at least 0.3 mm to the FN was aimed for. If preferred, the surgeon can further adapt the access path. Finally, the surgeon must confirm the planned access path.

**Figure 6:**
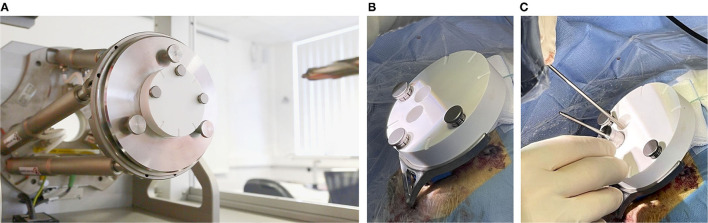
**(A)** The (white) blank jig inside of the automated manufacturing machine, and **(B)** mounted on the frame. **(C)** A tool guide is put into the main through-hole and the sham drill bit can be inserted into the tool guide. The second through-hole can be used to inspect the path inside the conventionally opened area.

To manufacture the patient-specific positioning jig based on previously-performed planning, the coordinates of the access path were wirelessly transferred to the fully automated manufacturing system. The machine consists of a high-precision hexapod, which is a parallel robot, and a linear axis with a drill unit in order to drill a through-hole into a blank jig. The position and orientation of the jig's through-hole represent the planned access path. Once the positioning jig is mounted onto the frame, this patient-specific through-hole aligns with the planning. After a production time of approximately 5 min (Figure 6A), the individualized positioning jig was taken out of the machine, measured with the coordinate measurement machine, disinfected, and steam sterilized. The sterilization was performed at the central sterilization facilities of the hospital in the routine process. After that, the individualized positioning jig was put in a sterile barrier system, labeled, and transported to the operating theater.

In the final step, the positioning jig was handed over to the surgeon, unpacked, and attached to the frame with the jig fasteners (Figure 6B). The tool guide was inserted into the through-hole of the positioning jig in order to guide the different sizes of sham drill bits. The sham drill bits and the tool guide were designed with a tight clearance fit (can only slide forward and backward with one degree of freedom) into the through-hole of the positioning jig. By this, they simulate different drill bits for the minimally-invasive approach for the previously planned access path. For the purpose of this trial, the mastoidectomy has already been performed in the meantime and the sham drill bits are just used to simulate the minimally-invasive access path for accuracy evaluation. For documentation reasons, the positioning jig contains two through-holes: the main through-hole, as described above, to guide the sham drill bit and an additional hole that can be used to visually inspect the situs under the positioning jig endoscopically or microscopically (Figure 6C).

After accuracy evaluation, our components were removed. Electrode array insertion and implant placement were performed as per our conventional techniques for CI surgery.

### 2.5. Evaluation Method

A conventional opening for CI implantation including mastoidectomy and posterior tympanotomy was performed while the mini-stereotactic frame was fixated on the participant's skull. In parallel, planning the access path and production of a patient-specific jig was performed. The primary outcome was a qualitative assessment of the workflow with regard to the planning, based on radiological image data, the production, and the attachment of the patient-specific jig to the mini-stereotactic frame. A secondary outcome was a subjective assessment of the fixation procedure and the resulting fixation strength of the mini-stereotactic frame. Instead of minimally-invasive drilling, a semi-quantitative evaluation of the overall accuracy was performed by inserting sham drill bits through a guidance through-hole in the patient-specific jigs (Figures 1A,C). Since the surgical site has already been completely opened and the facial recess has been exposed, we were able, with a surgical microscope or an endoscope, to assess and document the following binary properties:

- Does a sham drill bit point through the open space in the facial recess without touching any of the identified risk structures?
- What is the largest diameter of the sham drill bit that can be pointed through the facial recess?
- Does the 3 mm diameter shaft of the sham drill bit touch the external auditory canal (EAC) wall?

Diameters of 1.5, 1.8, 2.2, and 2.6 mm were tested. The smallest diameter was chosen to allow just enough space to insert a MED-EL Flex electrode lead with a maximal diameter of 1.3 mm. The 1.8 mm diameter was chosen because it has been reported by other research groups in this area. The accuracy of the jig manufacturing process was measured with a tactile coordinate measurement machine (8-axis FARO Quantum S / FaroArm V2, FARO Technologies, Inc.) and the effective deviations at the projected depth of the target point of the access path are computed.

### 2.6. Research Hypothesis

The hypothesis of this feasibility investigation: If the sham drill bit is

1. restricted in degrees of freedom along the planned path by guiding it through our patient-specific positioning jig, and
2. the tip points through the facial recess in the conventionally opened mastoid and posterior tympanotomy,

then it follows that

- the anatomical segmentations in the planning software,
- the planned path,
- the mini-stereotactic frame,
- the stability of bone fixation of the mini-stereotactic frame onto the skull,
- the accuracy of the X-ray marker and its automatic localization in the software,
- the jig manufacturing process,
- the attachment of the positioning jig to the frame, and
- the chosen diameter of said sham drill bit,

in combination, can be considered suitable for an MIS. The thickness of the bone layer above the chorda tympani and FN will be considered safe by surgeons based on the situation *in situ*.

## 3. Results

The CI surgeries were performed by three different surgeons between April and December 2021. No adverse or serious adverse events related to the trial occurred. The surgery, including the general anesthesia, was well tolerated by all patients. Temporary wound dehiscence in patient 01 could be attributed to reasons outside of the trial.

### 3.1. Frame Fixation

After performing skin incisions (3x about 5 mm for the three pins and one 10 mm in length for the bone screw), it was possible to remove the periost through small skin incisions at the positions of the three pins. The procedure for fixation of the frame, including pre-drilling and tightening the self-tapping bone screw was completed in, on average, 27 min (range 14–49 min). In two cases, however, the pre-drilling was attempted without using the frame to guide the direction of pre-drilling. This resulted in a hole too wide for the 2.0 mm bone screw. The fixation then had to be performed with a bone screw of the same length but with a larger diameter of 2.4 mm. Due to this, the fixation took a maximum of 49 min. The torque to tighten the bone screw and the mounting screw for attaching the frame could both be set with the torque ratchet to the predefined 30–35 Ncm. We subjectively confirmed a rigid fixation of the frame by carefully attempting to pull, move, and rotate the frame. In all cases, the frame remained rigidly fixated throughout the surgery.

### 3.2. Planning

The initial position of the frame was suitable in all cases and allowed an access path within the boundaries of the yet-to-be-created positioning jig. No repositioning of the frame was required. Due to the frame's symmetrical design, it was equally appropriate for left and right ears. The planning of a suitable access path with a diameter of 1.5 mm through the facial recess toward the round window of the cochlea was possible in all cases. Furthermore, the 0.3 mm margin to the FN and a margin to the EAC wall were possible to be executed. In case 01, the position of EAC did not allow a larger distance to the FN, whereas the distance to the CT was larger (0.86 mm). Table 1 summarizes the planned distances.

**Table 1:** Demographics, the width of the facial recess, distances from the planning, and results of the first six patients.

**Patient**	**01**	**02**	**03**	**04**	**05**	**06**
Age (years)	69	61	53	32	60	72
Sex	F	F	F	M	F	M
Side	L	L	L	L	R	R
1.5 mm sham drill bit passing?	Yes	Yes	Yes	Yes	Yes	Yes
1.8 mm sham drill bit passing?	Yes	Yes	No	Yes	No	No
EAC wall touched?	No^*^	No	No	No	No	No
Planned distance to EAC wall	0.6	0.7	1.9	1.8	0.9	1.3
Facial recess width (*d*_*FN*_ + *d*_*CT*_)	1.04+1.61	1.16+1.29	1.06+2.28	1.33+1.08	1.08+1.60	1.11+0.95
Planned margin to FN ($m_{FN}^{1.5}$)	0.29	0.41	0.31	0.58	0.33	0.36
Planned margin to CT ($m_{CT}^{1.5}$)	0.86	0.54	1.53	0.33	0.85	0.20
Deviation in jig	0.14	0.24	0.08	0.08	0.16	0.05
Bone thickness at screw	6.9	4.6	5.6	4.8	6.2	3.7

*The width of the facial recess is reported as two numbers: d_FN_ as the shortest effective distance from the central longitudinal axis of the planned path to the segmented facial nerve (FN) canal, and d_CT_ respectively for the chorda tympani (CT). The planned margins to these nerves are denoted as $m_{FN}^{1.5}$ and $m_{CT}^{1.5}$ for the 1.5 mm diameter sham drill bit. This description of the effective width of the facial recess allows computing the margins by subtracting the radius of the drill bit for the chosen position of the path, e.g., $m_{FN}^{1.5}=1.04\text{mm}-\left(1.5\text{mm}/2\right)=0.29\text{mm}$ for case 01. The planned distance to the external auditory canal (EAC) wall is measured from the border of the 3 mm diameter part of the planned path to the end of the bony wall. All numbers (except age) are given in mm*.

* *Additional thinning out the EAC wall was required*.

### 3.3. Jig Manufacturing and Sterilization

The wireless transfer of the planning data to the manufacturing system was successful in all cases. In that machine, a blank jig was mounted onto hexapod parallel kinematics. The manufacturing process of the patient-specific positioning jig is fully automated, and, as anticipated, took 5 min for each patient. The positioning jig was disinfected, sterilized, packaged in a sterile barrier system, and transported to the operating theater. Due to the large sterilizers at the clinic, the whole process of manual disinfection (5 min), sterilization (65 min), packaging (1 min), and transport (14 min) took 85 min on average. The accuracy of the manufacturing process of the patient-specific through-hole is reported in Table 1 as "Deviation in jig", meaning the deviations projected at the target point of the access path.

### 3.4. Sham Drill Bits

**Figure 7:**
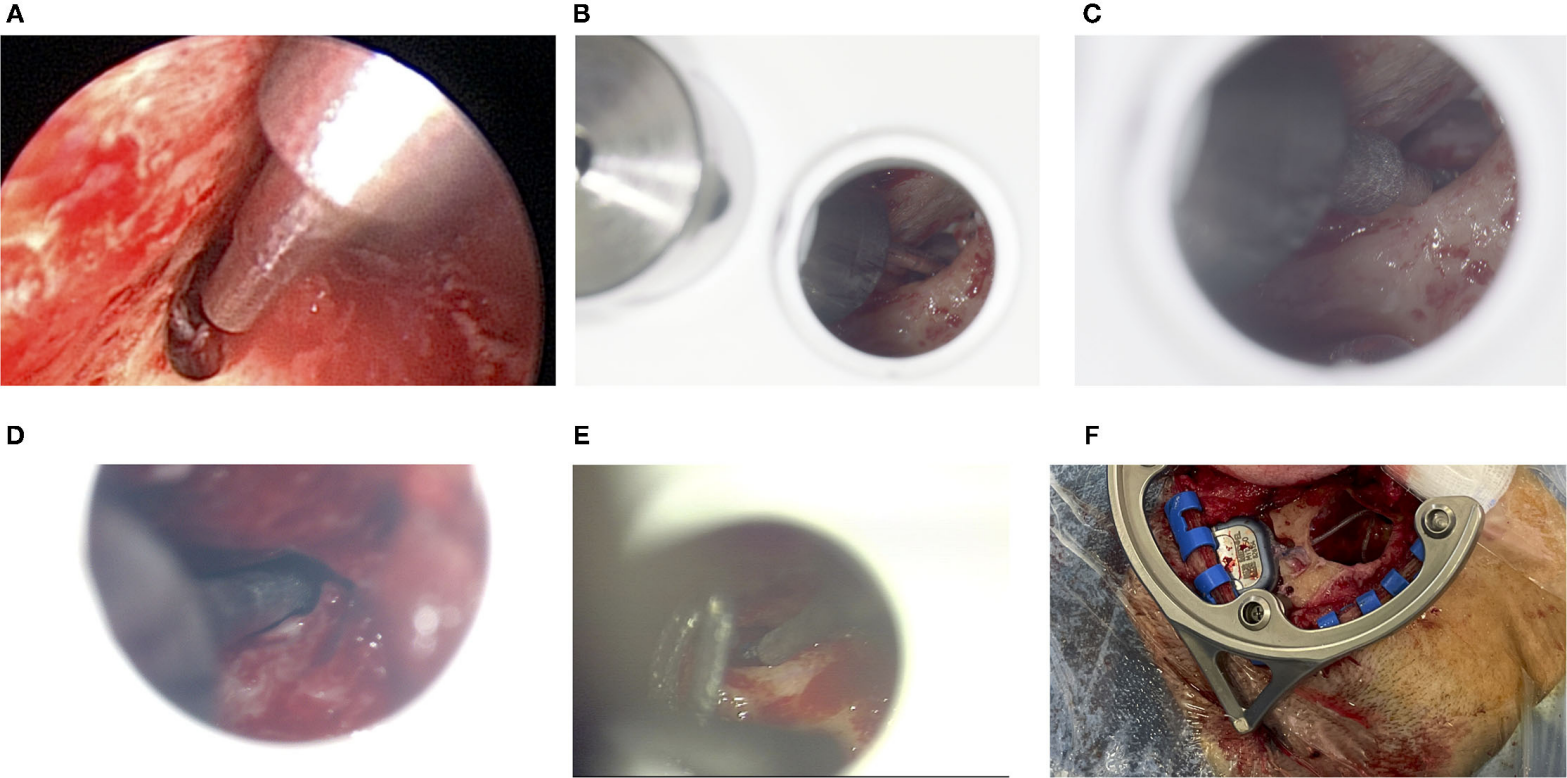
**(A)** Endoscopic inspection after the facial recess has conventionally been exposed for cochlear implant (CI) surgery for patient 01. The 1.8 mm sham drill bit points through the open facial recess without collisions. The large diameter in the top right of this image is 3 mm, which - in this case - comes close to the thinned out EAC. **(B)** Microscopic view for patient 03. The 1.5 mm sham drill bit fits through, whereas **(C)** the 1.8 mm sham drill bit does touch the bony layer above the FN. **(D)** For patient 04, the 1.8 mm sham drill bit passes through. **(E)** For patient 05, only the 1.5 mm sham drill bit just passes through very tightly. **(F)** The geometry of the frame allows making the bony implant bed while the frame remains fixated.

In all six cases, microscopic or endoscopic inspection verified that the sham drill bit successfully pointed through the posterior tympanotomy (Figure 7). While the 1.5 mm diameter of the sham drill bit could, in all cases, be passed freely through the conventionally-opened facial recess without any contact with bone, this was only possible in 3 of 6 cases for the 1.8 mm diameter of the sham drill bit. In the remaining 3 cases, the bony layer that was left above the FN was touched. In these cases, we visually assessed that the FN itself, running inside its bony canal, would most likely not have been mechanically damaged (refer to Table 1). The sham drill bit increases stepwise in diameter up to 3 mm. In all cases, it was possible to advance the sham drill bit without it touching the EAC wall. In case 01, however, additional thinning of the EAC wall with a diamond bur had to be performed.

### 3.5. Bone Thickness for Bone Screw

The screw is designed to reach 3.6 mm into the bone. As per the intraoperative CBCT scan, the bone thickness of the skull at the position of the bone screw was in mean 5.62 mm (range 3.7–6.9 mm), see last row of Table 1.

## 4. Discussion

In this article, we presented the promising results of the first six patients in this trial. These preliminary results strongly suggest that achieving high accuracy trajectories for future minimally-invasive drilling through the facial recess with this system seems both feasible and reproducible. Answering the research hypothesis this way: Any error in a) the planned access path, b) the anatomical segmentations, c) the mini-stereotactic frame and its bone fixation, d) the X-ray marker and its localization algorithm, e) the jig manufacturing process, or f) the attachment of the positioning jig would have led to not pointing through the facial recess or to directly touching risk structures by using a 1.5 mm sham drill bit.

All involved surgeons gave especially positive feedback on the fixation with a single bone screw. This is likely because it further simplifies the handling and provides a reliable bone fixation throughout the procedure. The Screw Implantation Safety Index (SISI) for 4 mm bone screws, introduced by Talon et al. (20), shows that our chosen location for the bone screw falls within the area of high safety.

In the facial recess approach to cochlear implantation, experienced surgeons almost always leave a thin layer of bone above the FN and CT. Sometimes this thin layer is even carefully removed with a diamond bur and the nerves are exposed. These aspects (exposure and thickness of the thin layer) are difficult to plan preoperatively with CT or CBCT imaging because a) only the bony structures are visible and b) the nerve has a smaller diameter than the nerve canal it runs through. For this reason, we intraoperatively compared if a drill path of a certain diameter would fit through the facial recess. Surprisingly, an access path with a diameter of 1.8 mm, which is a number often used in the minimally-invasive CI literature (8, 21–23), could in three of six cases not passed through the facial recess along the access path, even if the distance to the CT would have allowed a larger diameter. One reason for this is that the EAC wall limits the planning options for the access path. A second reason is that the effective width of the facial recess in the direction of the access path is often smaller (due to the projection) than the maximum anatomical width. The latter is reported by Jain et al. (24) as “The average maximum width of the FR was 2.93 ± 0.4 mm (range 2.24 − 3.45 mm) […]” and by Bielamowicz et al. (25) as 2.61 ± 0.70 mm. The diameters of sham drill bits larger than 1.8 mm could in no case be used to pass through the facial recess, therefore, we omitted them from Table 1. However, all cases could have been completed using a sham drill bit with a diameter of 1.5 mm. This seems in line with recent study by Auinger et al. (26), who wrote that "up to two thirds of ears were eligible for robotic cochlear implant surgery with the standard drill bit size of 1.8 mm" and "drill bit sizes ranging from 1.0 to 1.7 mm in diameter could increase feasibility up to 100%.” Labadie et al. (27) also used a smaller diameter of 1.59 mm to perform the drilling through the facial recess. Our trial, however, cannot make a statement about a possible deviation due to drilling into and through the mastoid bone because we only inserted sham drill bits. Future research, e.g., bench testing would benefit from providing this necessary evidence.

The minimally-invasive jig-based procedure described herein has the potential to reduce drilling and anesthesia time. By this, a completely new, practical, safe, and cost-effective alternative in CI care may be possible. Compared to other systems [i.e., Labadie et al. (13)], our aim was to use a stable fixation with only one single main central bone screw and to minimize the number of components that are assembled and attached to the patient. In our proposed system, the position and angular individualization are performed by drilling a through-hole in the corresponding angle into the blank jigs. We think this simplifies the surgical workflow and avoids use errors in the assembly. We propose an affordable alternative that is—compared to navigated, highly-specialized CI robotics—simpler but just as accurate.

The standardization of the operation, be it with the help of navigated robots or stereotactic frames, should allow CI implantation with greater accuracy and less variability in outcomes. CI recipients would likely benefit from the reduced trauma and a possibly shorter operation duration. This idea is becoming increasingly attractive in order to meet the rising need for "simpler" CI implantations in an aging population. In order to convince more CI candidates of the benefits of CIs use, the surgical procedure itself has to be made less invasive. A future application—where more evaluations will be required–could include difficult cases such as small or sclerotic mastoid or malposition of the FN.

## Data Availability Statement

The DICOM raw data cannot be made available due to data protection regulations. The other data presented in the study are included in the article, further inquiries can be directed to the corresponding authors.

## Ethics Statement

The studies involving human participants were reviewed and approved by Ethics Committee of MHH. The patients/participants provided their written informed consent to participate in this study.

## Author Contributions

RS is the lead investigator responsible for the trial. RS and TL and a third surgeon from the team performed the surgeries. SJ wrote this manuscript. JS and MK supported the OR team in this trial and developed the hardware prototypes. FR supported in path planning and programming. MF revised the manuscript. All authors contributed to the article and approved the submitted version.

## Funding

The presented study was funded in part by the Federal Ministry of Education and Research of Germany (BMBF, Grant Nos. 13GW0367A/B and 13GW0265A), and in part by European Union (EFRE) and by Lower Saxony (SER) ZW 3-85031593.

## Conflict of Interest

SJ, MK, and TL declare being limited partners of *HörSys IP GmbH* & *Co. KG* holds a financial stake in OtoJig GmbH, which is a German company that owns and further develops the described technology. SJ, FR, MK, and JS are employed by OtoJig GmbH. MF is employed at MED-EL, which holds a financial stake in OtoJig GmbH. The remaining author declares that the research was conducted in the absence of any commercial or financial relationships that could be construed as a potential conflict of interest.

## Publisher's Note

All claims expressed in this article are solely those of the authors and do not necessarily represent those of their affiliated organizations, or those of the publisher, the editors and the reviewers. Any product that may be evaluated in this article, or claim that may be made by its manufacturer, is not guaranteed or endorsed by the publisher.
